# Comparative analyses of the complete mitochondrial genomes of *Dosinia* clams and their phylogenetic position within Veneridae

**DOI:** 10.1371/journal.pone.0196466

**Published:** 2018-05-02

**Authors:** Changda Lv, Qi Li, Lingfeng Kong

**Affiliations:** 1 Key Laboratory of Mariculture, Ministry of Education, Ocean University of China, Qingdao, China; 2 Laboratory for Marine Fisheries Science and Food Production Processes, Qingdao National Laboratory for Marine Science and Technology, Qingdao, China; Institute of Oceanology, Chinese Academy of Sciences, CHINA

## Abstract

Mitochondrial genomes have proved to be a powerful tool in resolving phylogenetic relationship. In order to understand the mitogenome characteristics and phylogenetic position of the genus *Dosinia*, we sequenced the complete mitochondrial genomes of *Dosinia altior* and *Dosinia troscheli* (Bivalvia: Veneridae), compared them with that of *Dosinia japonica* and established a phylogenetic tree for Veneridae. The mitogenomes of *D*. *altior* (17,536 bp) and *D*. *troscheli* (17,229 bp) are the two smallest in Veneridae, which include 13 protein-coding genes, 2 ribosomal RNA genes, 22 tRNA genes, and non-coding regions. The mitogenomes of the *Dosinia* species are similar in size, gene content, AT content, AT- and GC- skews, and gene arrangement. The phylogenetic relationships of family Veneridae were established based on 12 concatenated protein-coding genes using maximum likelihood and Bayesian analyses, which supported that Dosininae and Meretricinae have a closer relationship, with Tapetinae being the sister taxon. The information obtained in this study will contribute to further understanding of the molecular features of bivalve mitogenomes and the evolutionary history of the genus *Dosinia*.

## Introduction

The genus *Dosinia* belonging to the family Veneridae in the superfamily Veneracea are important marine bivalves inhabiting from intertidal zone to subtidal zone along shallow coasts [1]. It was recognized as one of the significant taxons both in stratigraphy and chronology [2]. Therefore, the evolution of genus *Dosinia* deserves a careful investigation for understanding the bivalve evolution and diversity.

Along with morphological classification, molecular analyses are essential for evaluating the diversity of metazoans because of their high informativeness and accuracy [3, 4]. However, in contrast to the large number of morphological descriptions of *Dosinia*, there have been few attempts to perform molecular phylogenetic analysis in this genus so far [5–10]. Most present work involving *Dosinia* clams used different gene sequences (e.g., ITS2, H3, *rrnL*) to resolve the phylogenetic relationships at the level of Heterodont or Veneroida, nevertheless, the accuracy of phylogenies reconstructed from single gene sequences is low due to substitutional saturation of nucleotide and horizontal gene transfer [11, 12].

In the recent studies on phylogenetic analysis of bivalves, mitochondrial (mt) genome stands out to be considered a useful tool for population genetic and phylogenetic studies [3, 4, 13–18]. Owing to abundance of mitochondria in cells, maternal inheritance (except for doubly uniparental inheritance), lack of recombination, absence of introns, and higher evolutionary rates, mtDNA sequences are established as a molecular marker in comparative and evolutionary genomics, molecular evolution, population genetics, species identification, and phylogenetic relationships at various taxonomic levels [4, 16, 19, 20]. Meanwhile, the phylogenetic analysis based on complete mitogenome has also proved to enhance resolution and statistical confidence of inferred phylogenetic trees when compared with the small portions of mt sequence [21–24]. With the technological and methodological advances, the amplification and sequencing of complete mt genomes become routine [25]. Recently, the number of available complete mitogenome sequences has increased considerably. However for the *Dosinia* genus, only one mt genome is available (*Dosinia japonica*) to date [10], and more mitogenome sequences from various *Dosinia* species are required for further analysis. The mt genome of *D*. *japonica* contains 37 genes including 13 for protein subunits of oxidative phosphorylation enzymes (*cox1-3*, *cytb*, *atp6*, *atp8*, *nad1-6* and *nad4l*), 22 transfer RNA genes necessary for translating 13 proteins, and two ribosomal RNA genes (*rrnL* and *rrnS*) [10, 26].

In most metazoan phyla, mt genomes are conserved in length and content, and the gene arrangements are rarely changed [27]. However, in mollusks, especially bivalves, the gene arrangements appear to be dramatically variable in the major groups, even in the same family or genus [3, 4, 15, 18, 19, 28–30]. Currently, 13 Veneridae mitogenomes are available in the GenBank, which are sequenced from species within six genera, including *Paphia*, *Meretrix*, *Ruditapes*, *Saxidomus*, *Cyclina* and *Dosinia*. Comparing gene orders of *Ruditapes philippinarum*, *Paphia euglypta*, *Meretrix lamarckii*, and *Saxidomus purpuratus* of family Veneridae which were from different genus, the four mitogenomes show no identical gene arrangement either in whole mitogenomes or protein-coding genes. Also, the bivalves in certain genus such as *Crassostrea*, *Meretrix* show great genetic variations in length and gene organization [31–34]. Thus, considering the different characteristics of mitogenomes in different genus within Veneridae, and the variations of genomic organization which would provide a good way to study the evolutionary history of the mt genomes, it was necessary to investigate the mtDNA characteristics shared by *Dosinia* for clarifying their phylogenetic position [3, 13].

In the present study, we sequenced the complete mt genomes of *D*. *altior* (Deshayes, 1853) and *D*. *troscheli* (Lischke, 1873), and compared them with that of *D*. *japonica* [10]. The gene arrangements were compared among three *Dosinia* species, together with the other 12 Veneridae species. In addition, we also reconstructed the phylogenetic relationship of 15 Veneridae species based on the concatenated nucleotide sequences of 12 protein-coding genes to better understand of the phylogenetic relationships of *Dosinia* genus within Veneridae.

## Materials and methods

### Ethics statement

This study was conducted in accordance with general governmental regulations. Field sampling did not require specific permissions for the location, and no endangered or protected species were involved in the experiments of this study.

### Sample collection and DNA extraction

A single specimen was obtained from each of the two *Dosinia* bivalve. *D*. *altior* was collected from Qinzhou (Guangxi province of China) and preserved frozen at -80 °C in 2017. *D*. *troscheli* was collected from Beihai (Guangxi province of China) and preserved in 95% ethanol in 2014. Specimens were taxonomically identified based on shell morphology [35] and DNA barcoding sequence (*cox1* gene) from GenBank and Boldsystems. Total genomic DNA was extracted from adductor muscle following the standard phenol-chloroform procedure described by Li *et al*. [36] with some modifications. The DNA quality was checked on 1.0% agarose gel.

### PCR amplification and DNA sequencing

Short fragments of the genes *cox1*, *rrnL*, *rrnS*, *cytb*, *nad1* were amplified with the universal primers of LCO1490/HCO2198 [37], 16SF/16SR [38], G12SF2/G12SR6, GcobF2/GcobR6 and Gnad1F/Gnad1R5 [30]. Based on the sequences of above fragments and the complete mitogenome of *D*. *japonica* [10], long-PCR primers were designed and employed to amplify the entire mt genomes (Table 1).

**Table 1:** Primer sets for PCR amplification.

Species	Genomic region	Primer name	Sequence	Annealing
*D*. *altior*	*cox1—nad1*	cox1-nad1_AF	TTGAACTATCTACCCTCCGT	48 °C
		cox1-nad1_AR	AGCCGAATAAGGCACTCC	
*D*. *altior*	*nad1—cob*	nad1-cob-F	CGTTTCCGCTTTTAGGGTGT	56 °C
		nad1-cob-R	GCTTCCGCATCTCTTTCT	
*D*. *altior*	*cob—rrnL*	cob-rrnL-AF	TTAGTGGAGTGAATGTGG	53 °C
		cob-rrnL-AR	GTTATCCCTGCGGTAGTT	
*D*. *altior*	*nad5*	nad5-F	TCACATGAAAAGTTTATGGCAC	52 °C
		nad5-R	ACACTGGGCACAATTACTACAC	
*D*. *altior*	*rrnL—nad5*	rrnL-nad5-F	GGAAAGAATAAAGCAAAAACTACCG	56 °C
		rrnL-nad5-R	ACCACCCCTAATCCATCTCACC	
*D*. *altior*	*nad5—rrnS*	nad5- rrnS-F	ACCAAGAGGGCTCAGATTCC	54 °C
		nad5- rrnS-R	TCACGACCTACACTTTTCAGACC	
*D*. *altior*	*rrnS—cox1*	rrnS-cox1_AF	TACCGCCGTTGTAAATAGTCTTG	56 °C
		rrnS-cox1_AR	CATCTAATATCTTTCCAGGCATAGC	
*D*. *troscheli*	*cox1—nad1*	cox1-nad1_TF	GCAGCAGTATATTATTGGC	54 °C
		cox1-nad1_TR	CACTAACTCAGACTCTCCTTC	
*D*. *troscheli*	*nad1—cob*	nad1-cob-F	CGTTTCCGCTTTTAGGGTGT	56 °C
		nad1-cob-R	GCTTCCGCATCTCTTTCT	
*D*. *troscheli*	*cob—rrnL*	cob-rrnL_TF	ATTATTCTCCAGAAGTAAGCCAAGC	54 °C
		cob-rrnL_TR	ACTCCACCTACCAACACAACCC	
*D*. *troscheli*	*rrnL—nad4*	rrnL-nad4-F	AAGCAAAAACTACCGCAG	54 °C
		rrnL-nad4-R	TAAATCCCTCAAAGAACCG	
*D*. *troscheli*	*nad4—nad5*	nad4-nad5-F	TTCTTTAGTAAGGCACGG	50 °C
		nad4-nad5-R	ATCCATCTCACCCCAAT	
*D*. *troscheli*	*nad5 –nad6*	nad5-nad6-F	ATGTGTGTGGGTGGTGTAAT	52 °C
		nad5- nad6-R	CACTCAACTCAATCGCTTTC	
*D*. *troscheli*	*nad6—rrnS*	nad6-rrnS-F	GATTGAGTTGAGTGAGGGGT	50 °C
		nad6- rrnS-R	TTCTATTAGCATTACTATGTTACG	
*D*. *troscheli*	*rrnS—cox1*	rrnS-cox1_TF	TAAGGTAAGTCGTAACATAGT	48 °C
		rrnS-cox1_TR	AACCCCAAACATAAACAG	

PCR reactions were performed in a 10 μl volume containing about 50 ng template DNA, 1× reaction Buffer (Mg^2+^ plus, Takara), 0.02 mM of each dNTP, 1 μM of each primer, 0.25 U LA-*Taq* polymerase (Takara). The long-PCR reactions for long fragments refer to Yuan *et al*. [18] protocols: an initial denaturation for 3 min at 94 °C, followed by 35 cycles comprising denaturation at 94 °C for 30 s, annealing at 48–56 °C for 30 s and extension at 68 °C for 5 min, the whole process was completed with a final extension at 72 °C for 10 min.

PCR products were purified with EZ-10 spin column DNA gel extraction kit (Sangon Biotech), and sequenced with the primer walking method directly. The sequencing was conducted on an ABI PRISM 3730 (Applied Biosystems) automatic sequencer in Beijing Genomics Institute (BGI) using standard Sanger sequencing chemistry.

### Sequence analysis and gene annotation

The DNA sequences obtained by sequencing the PCR-amplified DNA fragments were analyzed and assembled using Seqman program from DNASTAR (available at: http://www.dnastar.com/). Protein coding genes were annotated by using NCBI ORF Finder (https://www.ncbi.nlm.nih.gov/orffinder/) and BLASTx with the invertebrate mitochondrial code. The positions of tRNA genes were determined by ARWEN v.1.2 [39] and MITOS Web Server [40]; and secondary structures of tRNAs were inferred using MITOS in default search mode. The rRNA genes were identified by BLAST searches (http://www.ncbi.nlm.nih.gov/BLAST/), the boundaries of each gene were determined with multiple alignments of other 13 published bivalve mitogenomes from NCBI website. Repeat sequence patterns in the major no-coding region (MNR) were checked using the web-based software server Tandem Repeat Finder 4.0 (http://tandem.bu.edu/trf/trf.html) [41]. Mitogenome maps were drawn using the CGview Server (http://stothard.afns.ualberta.ca/cgview_server/) [42]. The two mitogenomes have been deposited in the GenBank database under the accession numbers MG543473 for *D*. *altior* and MG543474 for *D*. *troscheli*.

The base composition and skewness analyses were performed and compared between *D*. *altior* and *D*. *troscheli*, as well as *D*. *japonica*. The A+T content values and nucleotide frequencies were computed using Editseq program from DNASTAR. The GC- and AT- skews were calculated according to the formulae by Perna *et al*. [43]: AT-skew = (A -T)/(A + T); GC-skew = (G-C)/(G+C), where A, T, G and C are the occurrences of the four nucleotides. Codon usage analysis was performed by MEGA 6 [44].

### Phylogenetic analyses

Along with mitogenome sequences of *D*. *altior* and *D*. *troscheli*, all currently available mitogenomes of Veneridae including *R*. *philippinarum* (AB065375), *Meretrix petechialis* (EU145977), *Meretrix lyrata* (KC832317), *Meretrix lusoria* (GQ903339), *Meretrix meretrix* (GQ463598), *M*. *lamarckii* (GU071281), *P*. *euglypta* (GU269271), *Paphia textile* (JF969277), *Paphia amabilis* (JF969276), *Paphia undulata* (JF969278), *S*. *purpuratus* (KP419933), *Cyclina sinensis* (KU097333), and *D*. *japonica* (MF401432) were used in phylogenetic analysis. *Solen grandis* (HQ703012) and *Solen strictus* (JN786377) from the family Solenidae were used as outgroups. The phylogenetic relationships were reconstructed based on nucleotide sequences of 12 PCGs (except *atp8*). The nucleotide sequences were aligned with Clustal X [45] using default settings, followed by manual correction.

Phylogenetic trees were reconstructed using maximum-likelihood (ML) analysis and Bayesian inference (BI) approaches. For phylogenetic analyses based on nucleotide data, the most appropriate model GTR + G was selected by jMODELTEST using the Akaike Information Criterion (AIC). The ML analysis was conducted with PhyML 3.0 (http://www.atgc-montpellier.fr/phyml/) and 1000 bootstraps were used to assess the support of nodes. BI was performed using MrBayes 3.1.2 [46]. In the case of the Bayesian analysis, the Markov Chain Monte Carlo were run for 100,0000 generations with trees sampled every 100 generations. Stationarity was defined as mean standard deviation of split frequency less than 0.01.

## Results and discussion

### Genome organization

The complete mt genomes of *D*. *altior* and *D*. *troscheli* are 17,536 bp and 17,229 bp in length, respectively, and the length of *D*. *troscheli* mitogenome is the smallest among the available mitogenomes of Veneridae currently. Length differences are mostly due to the size variation of the non-coding region. In the *D*. *altior* mitogenome, there are 22 non-coding regions with a total of 2385 bp long varying from 1 bp to 1865 bp, and the *D*. *troscheli* mitogenome contains 21 non-coding regions with a total of 2140 bp with various lengths of 2–1521 bp. The longest non-coding region of two *Dosinia* mitogenomes are both located between *trnI* and *trnD*.

**Fig 1:**
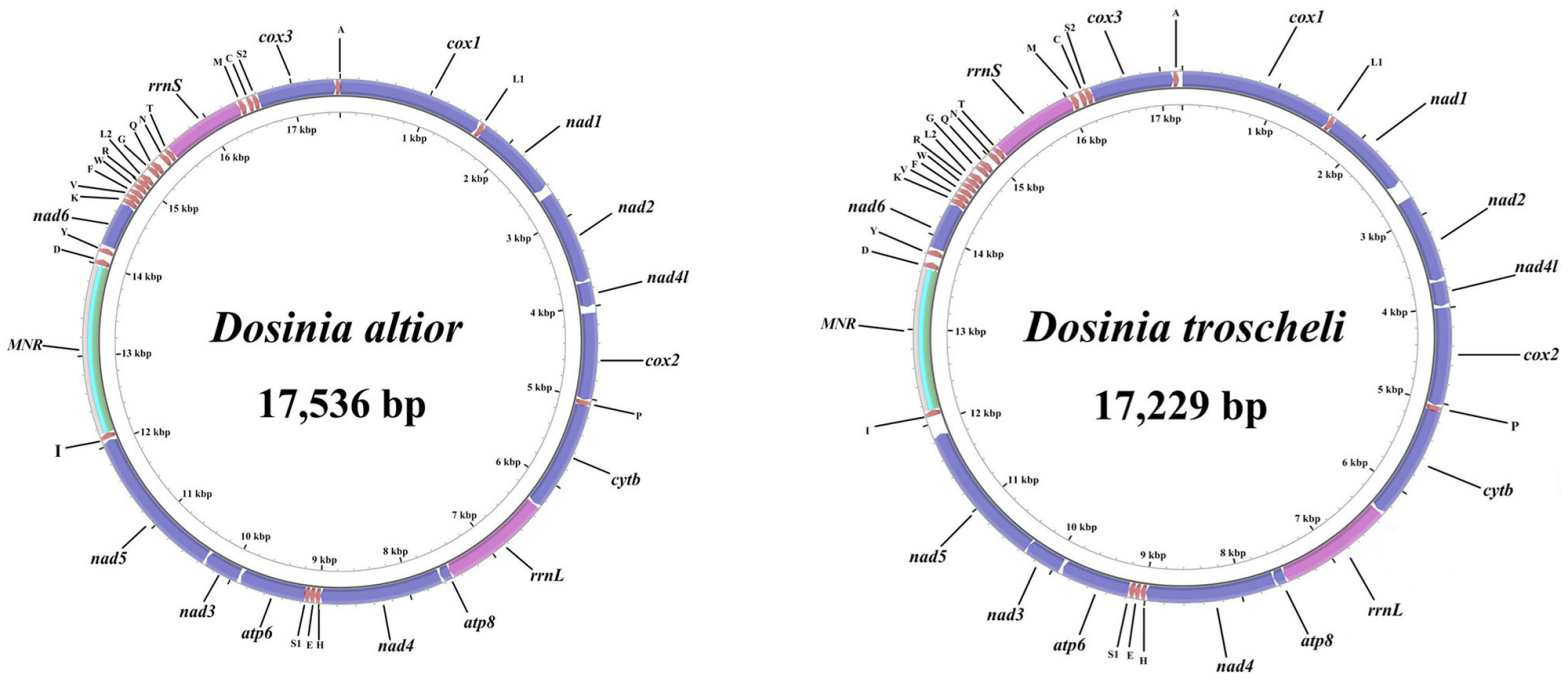
The organization of the mt genomes of *Dosinia altior* and *D*. *troscheli*. Genes for proteins and rRNA (*rrnS* and *rrnL*) are listed under abbreviations. The largest non-coding region is designated as MNR.

Both *D*. *altior* and *D*. *troscheli* mitogenomes contain 13 protein-coding genes (PCGs), 22 transfer genes and 2 ribosomal RNA genes (Fig 1). Main structure features of three *Dosinia* mitogenomes are summarized in Table 2. All the genes are encoded on the (+) strand. As a whole, although gene organization is known to vary extensively, even among species from the same genus [30], the three *Dosinia* mitogenomes showed a same gene order within the genus.

**Table 2:** Main structural features of the three mt genomes in *Dosinia* clams.

	*Dosinia altior*	*Dosinia troscheli*	*Dosinia japonica*
**T**otal size	17536	17229	17693
**A**+T%	69.57	69.67	69.97
*cox1*	1614(ATT/ TAG)	1614(TTG/ TAG)	1608(GTG/ TAG)
*trnL*^*CUN*^	65	65	66
*nad1*	930(TTG/ TAG)	927(ATA/ TAG)	906(ATG/ TAG)
*nad**2***	1020(GTG/ TAA)	1020(GTG/ TAA)	1020(GTG/ TAA)
*nad**4**l*	267(TTG/ TAA)	273(ATA/TAG)	276(ATA/ TAA)
*cox2*	954(ATT/ TAG)	1041(TTG/ TAA)	1011(GTG/ TAG)
*trnP*	65	65	65
*cytb*	1209(ATT/ TAA)	1212(ATT/ TAA)	1191(ATT/ TAA)
*rrnL*	1200	1202	1203
*atp**8***	114(GTG/ TAA)	114(ATG/ TAA)	114(ATG/ TAA)
*nad**4***	1359(ATA/ TAA)	1359(ATA/ TAG)	1359(ATG/ TAG)
*trnH*	63	62	62
*trnE*	65	61	61
*trnS* ^*UCN*^	63	63	63
*atp6*	741(ATT/ TAA)	729(ATG/ TAA)	729(ATG/ TAA)
*nad**3***	405(GTG/ TAA)	405(GTG/ TAA)	405(GTG/ TAA)
*nad**5***	1704(ATC/ TAA)	1527(ATA/ TAA)	1503(ATA/ TAA)
*trnI*	65	66	65
*trnD*	68	66	66
*trnY*	62	64	62
*nad6*	501(ATT/ TAG)	507(ATG/ TAA)	486(ATG/ TAA)
*trnK*	69	69	66
*trnV*	64	64	62
*trnF*	62	62	62
*trnW*	63	63	63
*trnR*	64	61	61
*trnL*^*UU**R***^	66	66	65
*trnG*	62	62	62
*trnQ*	69	69	67
*trnN*	67	65	67
*trnT*	62	62	62
*rrnS*	900	903	902
*trnM*	68	68	68
*trnC*	62	64	64
*trnS* ^*AGN*^	65	67	67
*cox**3***	870(ATG/ TAG)	870(ATG/ TAG)	870(ATG/ TAG)
*trnA*	63	62	62

AT content, AT-skew and GC-skew for mitochondrial sequences of two *Dosinia* species were shown in Table 3. The overall AT content of the *D*. *altior* mt genome is 69.57%, which is little lower than 69.67% of *D*. *troscheli* and 69.97% of *D*. *japonica*. Similar patterns appear in NCRs (72.45% *vs*. 74.07% *vs*.74.56%), tRNAs (72.04% *vs*. 72.72% *vs*. 72.23%), and *rrnL* (73.08% *vs*. 73.27% *vs*. 74.15%). In order to evaluate the base bias in the mitogenomes, we measured AT-skew and GC-skew in different gene regions of *D*. *altior* and *D*. *troscheli*, and compared with *D*. *japonica*. The results indicated that the values of AT-skew of three species were mostly negative except *rrnS*, as well as GC-skew values were all positive. Furthermore, the AT-and GC-skewness of entire mtDNA indicates the occurrence of more T than A and more G than C in *Dosinia* genus (AT-skew, -0.20 and -0.19; GC-skew, 0.43 and 0.41).

**Table 3:** AT content, AT-skew and GC-skew for mitochondrial genes of *D*. *altior*, *D*. *troscheli* and *D*. *japonica*.

Feature	(A+T) %			AT-skew			GC-skew		
	*D*. *altior*	*D*. *troscheli*	*D*. *japonica*	*D*. *altior*	*D*. *troscheli*	*D*. *japonica*	*D*. *altior*	*D*. *troscheli*	*D*. *japonica*
**Genome**	69.57	69.67	69.97	-0.20	-0.19	-0.19	0.43	0.41	0.39
**PCGs**	68.21	68.06	68.20	-0.29	-0.27	-0.27	0.42	0.39	0.38
**NCRs**	72.45	74.07	74.56	-0.02	-0.06	-0.08	0.60	0.59	0.55
**tRNAs**	72.04	72.72	72.23	-0.09	-0.06	-0.07	0.37	0.35	0.34
***rrnL***	73.08	73.27	74.15	-0.06	-0.06	-0.05	0.42	0.42	0.38
***rrnS***	71.44	70.76	69.18	0	0	0	0.30	0.32	0.30
***cox1***	65.68	65.99	65.49	-0.27	-0.26	-0.25	0.29	0.27	0.25
***nad1***	67.20	65.91	65.89	-0.28	-0.32	-0.29	0.44	0.44	0.35
***nad2***	68.82	68.73	68.92	-0.29	-0.26	-0.28	0.60	0.55	0.54
***nad4l***	75.28	71.79	72.83	-0.40	-0.39	-0.36	0.58	0.56	0.60
***cox2***	67.92	67.72	67.56	-0.17	-0.08	-0.12	0.50	0.51	0.52
***cytb***	65.84	66.09	66.41	-0.33	-0.31	-0.31	0.25	0.22	0.23
***atp8***	70.18	71.05	69.30	-0.52	-0.48	-0.57	0.24	0.09	0.26
***nad4***	69.09	69.76	69.54	-0.30	-0.27	-0.28	0.50	0.43	0.41
***atp6***	69.91	71.06	71.06	-0.32	-0.29	-0.29	0.26	0.25	0.25
***nad3***	68.89	69.38	69.38	-0.33	-0.32	-0.32	0.52	0.53	0.53
***nad5***	69.48	69.16	69.73	-0.24	-0.25	-0.23	0.48	0.41	0.38
***nad6***	70.46	70.02	72.22	-0.32	-0.32	-0.28	0.62	0.57	0.57
***cox3***	67.47	66.21	66.44	-0.30	-0.30	-0.31	0.36	0.37	0.36

### Protein-coding genes

Both *D*. *altior* and *D*. *troscheli* mt genomes encode the full set of 13 proteins. The whole size of the PCGs of *D*. *altior* was 11,688 bp, higher than the length of *D*. *troscheli* which is 11,598 bp. The overall AT content of the 13 PCGs was 68.21% in the *D*. *altior* mitogenome, ranging from 65.68% (*cox1*) to 75.28% (*nad4l*), and in *D*. *troscheli* mitogenome, the overall AT content of 13 PCGs was 68.06%, which is ranging from 65.91% (*nad1*) to 71.79% (*nad4l*).

**Fig 2:**
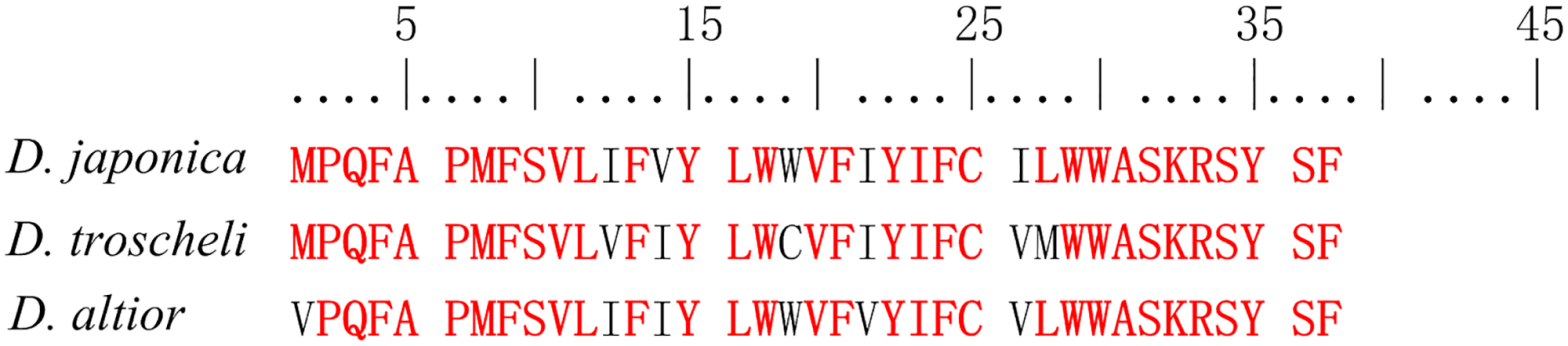
Alignment of *atp8* sequences of *D*. *japonica*, *D*. *troscheli* and *D*. *altior*.

In the 13 PCGs identified in *Dosinia* mitogenomes, we found the *atp8* which has been reported as missing in several bivalve species. Likewise, there are also many accurate searches referring to the identification of this gene, so the alleged lack of *atp8* within some bivalve species is likely due to inaccurate annotation due to the extreme variability and the small size of the gene in most cases [4, 13, 15, 18]. The *atp8* was found in publicly available mitogenome sequences of Veneridae species (Table 4). In genus *Dosinia*, this short gene encoded a 38 amino acids protein starting with ATG and GTG (ATG in *D*. *troscheli* and *D*. *japonica*, GTG in *D*. *altior*), and end with a stop codon (TAA). The location of the *atp8* within the mitogenomes is same in the three species of *Dosinia* genus, which is between *rrnL* and *nad4*. Amino acid sequences of these ATPases were aligned among three *Dosinia* clams, and the conversed motifs were found, in particularly, the N- terminus and the C- terminus (Fig 2).

**Table 4:** Presence of the *atp8* in the mitogenomes of the Veneridae family.

Species	From	To	Start	Stop	Length	aa	Reference
*Ruditapes philippinarum*	5968	6087	ATT	TAG	120	40	[4]
*Meretrix petechialis*	8532	8672	ATA	TAG	141	47	[4]
*Meretrix lamarckii*	8835	8954	ATG	TAA	120	40	[4]
*Meretrix lyrata*	8753	8872	ATG	TAG	120	40	[4]
*Meretrix lusoria*	8642	8761	ATG	TAG	120	40	[4]
*Meretrix meretrix*	8532	8672	ATA	TAG	141	47	[4]
*Paphia euglypta*	12994	13110	ATA	TAA	117	39	[4]
*Paphia textile*	13019	13132	ATG	TAA	114	38	[4]
*Paphia amabilis*	14035	14148	ATG	TAG	114	38	[4]
*Paphia undulata*	12642	12755	ATG	TAA	114	38	[4]
*Saxidomus purpuratus*	9557	9673	ATG	TAA	117	39	[4]
***Cyclina sinensis***	8568	8684	ATG	TAA	117	39	[4]
***Dosinia japonica***	7567	7680	ATG	TAA	114	38	[10]
***Dosinia altior***	7514	7627	GTG	TAA	114	38	This study
***Dosinia troscheli***	7523	7636	ATG	TAA	114	38	This study

Most of the PCGs in the three *Dosinia* mitogenomes initiate with conventional start codons ATN (ATT, n = 7; ATA, n = 7; ATG, n = 11). However, an alternative initiation codon TTG (n = 4) was detected in four genes *(nad1*, *nad4l*, *cox1*, and *cox2*), GTG (n = 9) was detected in *cox1*, *cox2*, *nad2*, *nad3* and *atp8* genes, and ATC (n = 1) in the *nad5* gene of *D*. *altior*. Genes starting with TTG, GTG and ATC were also found in mitogenomes of other Veneridae species such as *M*. *petechialis* and *M*. *meretrix* (*cox1*: TTG), *P*. *textile* (*nad1*: GTG) and *M*. *lamarckii* (*nad1*: ATC). As for termination codons, all 13 PCGs of the three mt genomes end in a full stop codon, either TAA (n = 24) or TAG (n = 15). There are four genes employing the same initiation and termination codons in *D*. *altior*, *D*. *troscheli* and *D*. *japonica* (*nad2*: GTG/TAA, *cytb*: ATT/TAA, *nad3*: GTG/TAA, *cox3*: ATG/TAG).

Codon usages of three *Dosinia* species were presented in Table 5. All stop codons were excluded from the calculation. The mt genome of *D*. *altior* encoded 3883 amino acids in total, compared with 3853 in *D*. *troscheli* and 3813 in *D*. *japonica*, respectively. All codons are used in three mitogenomes but with different frequencies. The three predominant codon families are UUU (phenylalanine), UUA (leucine) and GUU (valine) in *D*. *altior* mitogenome, and there are UUU (phenylalanine), UUA (leucine) and AUU (isoleucine) in *D*. *troscheli*, as all of these codons are A+T-rich codon families. On the contrary, the least chosen codons are from G+C-rich codon families such as CGC (arginine) and CCC (proline).

**Table 5:** Codon usages of the protein-coding genes in *D*. *altior*, *D*. *troscheli* and *D*. *japonica* mt genomes.

Amino acid	Code	N(RSCU)			Amino acid	Code	N(RSCU)		
		*D*. *altior*	*D*. *troscheli*	*D*. *japonica*			*D*. *altior*	*D*. *troscheli*	*D*. *japonica*
F	UUU	353(1.81)	351(1.8)	353(1.8)	Y	UAU	139(1.84)	133(1.77)	128(1.7)
	UUC	38(0.19)	40(0.2)	40(0.2)		UAC	12(0.16)	17(0.23)	23(0.3)
L	UUA	294(3.15)	293(3.21)	293(3.27)	H	CAU	59(1.87)	55(1.72)	49(1.61)
	UUG	164(1.76)	147(1.61)	134(1.5)		CAC	4(0.13)	9(0.28)	12(0.39)
L	CUU	57(0.61)	60(0.66)	67(0.75)	Q	CAA	26(1)	22(0.86)	24(0.98)
	CUC	4(0.04)	2(0.02)	7(0.08)		CAG	26(1)	29(1.14)	25(1.02)
	CUA	28(0.3)	32(0.35)	26(0.29)	N	AAU	104(1.94)	101(1.74)	84(1.62)
	CUG	13(0.14)	13(0.14)	10(0.11)		AAC	3(0.06)	15(0.26)	20(0.38)
I	AUU	200(1.83)	211(1.9)	212(1.93)	K	AAA	70(1.12)	78(1.31)	79(1.33)
	AUC	19(0.17)	11(0.1)	8(0.07)		AAG	55(0.88)	41(0.69)	40(0.67)
M	AUA	167(1.39)	158(1.39)	162(1.36)	D	GAU	81(1.88)	79(1.82)	76(1.79)
	AUG	73(0.61)	70(0.61)	76(0.64)		GAC	5(0.12)	8(0.18)	9(0.21)
V	GUU	219(1.93)	194(1.75)	209(1.96)	E	GAA	58(1.08)	55(0.99)	61(1.12)
	GUC	9(0.08)	14(0.13)	9(0.08)		GAG	49(0.92)	56(1.01)	48(0.88)
	GUA	122(1.98)	140(1.26)	125(1.17)	C	UGU	83(1.84)	83(1.89)	81(1.84)
	GUG	103(0.91)	95(0.86)	83(0.78)		UGC	7(0.16)	5(0.11)	7(0.16)
S	UCU	111(2.21)	100(1.96)	102(2.0)	W	UGA	55(0.99)	63(1.18)	51(0.96)
	UCC	11(0.22)	20(0.39)	9(0.18)		UGG	56(1.01)	44(0.82)	55(1.04)
	UCA	30(0.6)	35(0.68)	37(0.72)	R	CGU	35(2.15)	32(2.03)	33(2.1)
	UCG	17(0.34)	10(0.2)	20(0.39)		CGC	1(0.06)	2(0.13)	1(0.06)
P	CCU	71(2.47)	69(2.4)	56(2.0)		CGA	13(0.8)	9(0.57)	18(1.14)
	CCC	1(0.03)	5(0.17)	12(0.43)		CGG	16(0.98)	20(1.27)	11(0.7)
	CCA	24(0.83)	26(0.9)	23(0.82)	S	AGU	82(1.63)	77(1.51)	83(1.62)
	CCG	19(0.66)	15(0.52)	21(0.75)		AGC	6(0.12)	15(0.29)	15(0.29)
T	ACU	79(2.75)	70(2.55)	70(2.46)		AGA	91(1.81)	92(1.8)	103(2.01)
	ACC	6(0.21)	7(0.25)	8(0.28)		AGG	54(1.07)	60(1.17)	40(0.78)
	ACA	19(0.66)	20(0.73)	26(0.91)	G	GGU	111(1.54)	106(1.5)	103(1.47)
	ACG	11(0.38)	13(0.47)	10(0.35)		GGC	8(0.11)	12(0.17)	14(0.2)
A	GCU	92(2.59)	101(2.69)	99(2.66)		GGA	79(1.09)	81(1.15)	70(1)
	GCC	6(0.17)	4(0.11)	5(0.13)		GGG	91(1.26)	83(1.18)	93(1.33)
	GCA	22(0.62)	26(0.69)	30(0.81)					
	GCG	22(0.62)	19(0.51)	15(0.4)					

N is representative for the total number of particular codon in all protein-coding genes.

RSCU is representative for the relative synonymous codon usage.

### Transfer RNA and ribosomal RNA genes

*D*. *altior* and *D*. *troscheli* mitogenomes contain the usual set of 22 tRNA genes typical of metazoans, two copies of *trnS* and *trnL*, and one tRNA gene for each of the other 18 amino acids. All the tRNA genes are encoded on the (+) strand. The tRNA genes are interspersed in the mt genome, and ranged from 61 bp (*trnE* and *trnA*) to 69 bp (*trnK* and *trnQ*). There are seven tRNA genes (*trnS*, *trnP*, *trnF*, *trnW*, *trnG*, *trnT*, *trnM*) which have identical lengths within the three species from *Dosinia* (Table 2). The predicted secondary structures of tRNAs in two *Dosinia* clams were shown in S1 and S2 Figs. The majority of tRNAs can be folded into typical clover-leaf secondary structures except for *trnS*^*AGN*^ and *trnS*^*UCN*^, which lack the DHU arm. However, absence of the DHU arm in the secondary structure of *trnS*^*AGN*^ and *trnS*^*UCN*^ is commonly observed in molluscs [33, 47, 48]. The *trnY* of *D*. *altior* showed no terminal TψC loop, which have previously been found in mtDNA of other bivalve species, such as genus *Donax* [4]. The accepter stem and anticodon stem of tRNAs in *Dosinia* mt genomes are 5–8 bp, while the dihydrouracil stem and TψC stem are 3–5 bp in length.

The *rrnL* and *rrnS* of *D*. *altior* and *D*. *troscheli* were identified by sequence comparison with the available 13 Veneridae species from GenBank, though an accurate delimitation of gene boundaries must await transcript mapping. All *rrnS* in *Dosinia* mt genomes were located between *trnT* and *trnM*, which was same with that in *S*. *purpuratu*s and *C*. *sinensis*. The length of *rrnS* in three mt genomes ranged from 900 bp (*D*. *altior*) to 903 bp (*D*. *troscheli*), with A+T content between 69.18% (*D*. *japonica*) and 71.44% (*D*. *altior*). On the other hand, the *rrnL* was flanked by *cytb* and *atp8* in all *Dosinia* mt genomes, just like in *M*. *lyrata* [34], *M*. *lusoria* [33], and *M*. *lamarckii* [32]. Its size varied from 1,200 bp (*D*. *altior*) to 1,203 bp (*D*. *japonica*), and A+T content ranged between 73.08% (*D*. *altior*) and 74.15% (*D*. *japonica*). In the mt genome of *D*. *altior*, the size of either *rrnS* or *rrnL* is the shortest within that of mitogenomes in the family Veneridae [10, 27, 30, 32–34].

### Non-coding regions and repeat units

Differences of genome size are mainly due to different lengths of non-coding regions (NCRs) [30]. In the comparison of NCRs within the three mt genomes of genus *Dosinia* (Table 6), the mt genome of *D*. *altior* contained 23 NCRs ranging in size from 1 to 1865 bp, and the total length was 2385 bp, which was longer than *D*. *troscheli*. *D*. *troscheli* mitogenome contained 21 NCRs ranging in size from 2 to 1521 bp, and the whole length of that was 2140 bp. Both lengths of NCRs in *D*. *altior* and *D*. *troscheli* are shorter than that of *D*. *japonica*. Within these non-coding sequences, a 1521 bp and a 1865 bp nucleotide segments were putatively identified as the major non-coding region (MNR) of *D*. *altior* and *D*. *troscheli*. The MNR has a high A+T content of 71.26% in *D*. *altior* and 72.45% *D*. *troscheli*, which is higher than the average of the whole genome (69.57% in *D*. *altior*, 69.67% in *D*. *troscheli*) and PCGs (68.21% in *D*. *altior*, 68.06% in *D*. *troscheli*). The increased A+T content of MNR is thought to contain the signals for replication and transcription, and hence is referred to as the control region [49].

**Table 6:** Comparison of non-coding regions within the three mt genomes of genus *Dosinia*.

species	No. of NCR	Total length (bp)	Proportion of mitogenome (%)	Length of MNR (bp)	A+T% of MNR
***D***. *altior*	23	2385	13.6	1865	71.26
***D***. *troscheli*	21	2140	12.4	1521	72.45
***D***. *japonica*	24	2708	15.3	2027	72.92

**Fig 3:**
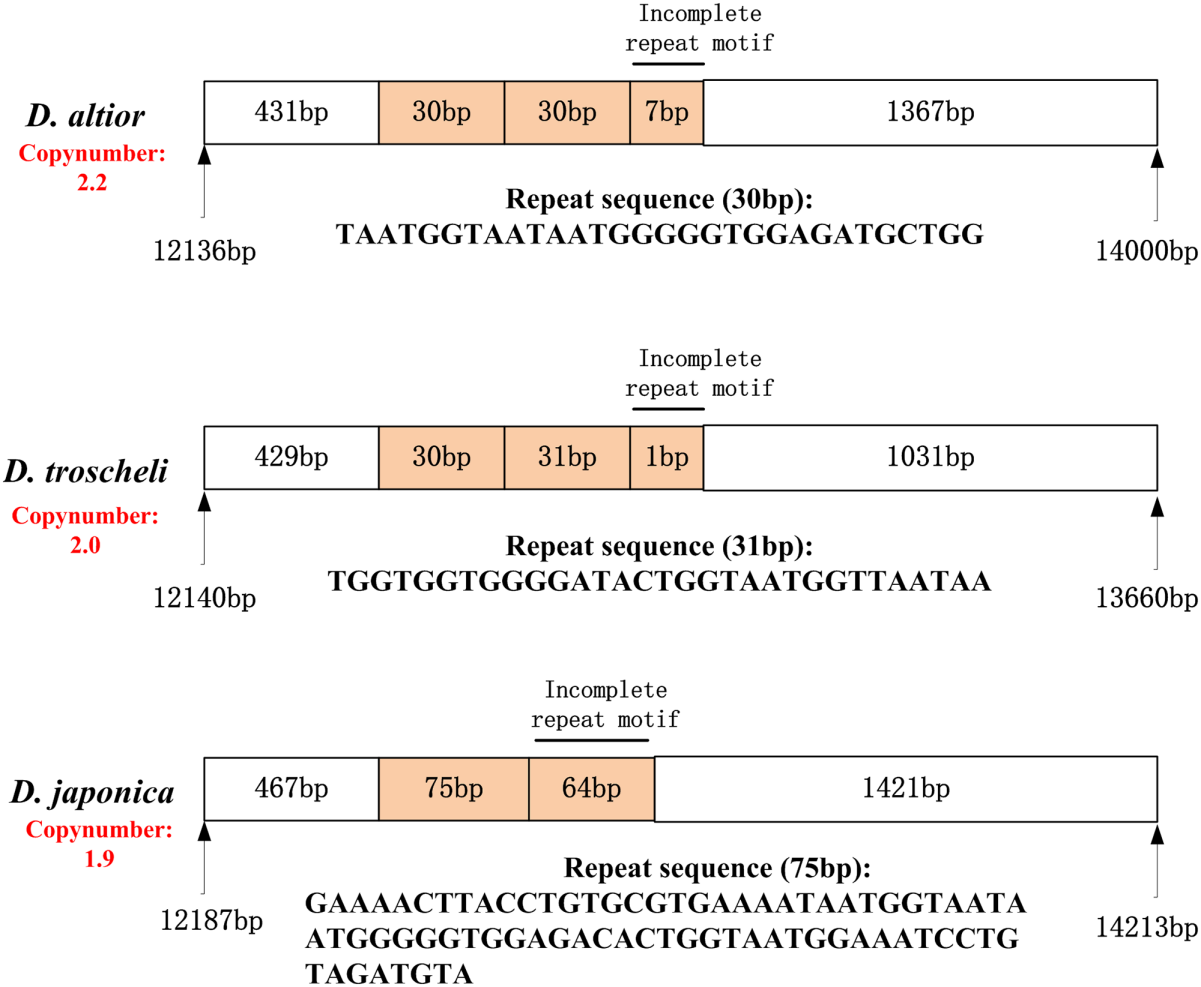
The structure of MNR and the tandem repeats elements of *D*. *altior*, *D*. *troscheli*, *D*. *japonica*. The orange sections indicate the tandem repeat regions.

In addition, the MNR has been regarded as the most easily relocated segment followed by tRNA genes and PCGs [25, 50, 51]. For example, the gene arrangements of four *Paphia* genomes are identical, but locations of MNRs are obviously different [30]. However, in the present study, we observed that the MNRs of the three *Dosinia* clams were all located between *trnI* and *trnD* genes, although with different tandem repeats. Tandem repeats have been described in the non-coding regions of metazoan [52–55], and in family Veneridae, the tandem repeat units were regarded as a common feature for mitogenomes, such as in *M*. *petechialis*, *M*. *lusoria*, and *R*. *philippinarum* [56]. The tandem repeat units found in *D*. *altior*, *D*. *troscheli* and *D*. *japonica* were all occurring around about 450 bp from the 5’ end of the non-coding region (Fig 3). There were 2.2 nearly identical copies of a 30 bp unit in the MNR of *D*. *altior*, and 2 nearly identical copies of a 30 bp sequence in *D*. *troscheli*, both the total lengths were shorter than that of *D*. *japonica*. *D*. *japonica* contained 1.9 copies of 75 bp repeat motif in MNR. Each tandem repeat motif of MNR in the *Dosinia* clams could form a secondary structure with a stem-loop when the sequence is folded to minimize the free energy of the structure. Great divergence in the tandem repeat region of different species which were closely related, even from same genus, was common, such as the *M*. *lusoria* and *M*. *petechialis* from *Meretrix* genus [33]. Further study of tandem repeats in the control region is needed, as it is important to illuminate a variety of process, including the molecular mechanisms for their generation and their possible functional implications [57].

### Gene arrangement and phylogenetic analyses

**Fig 4:**
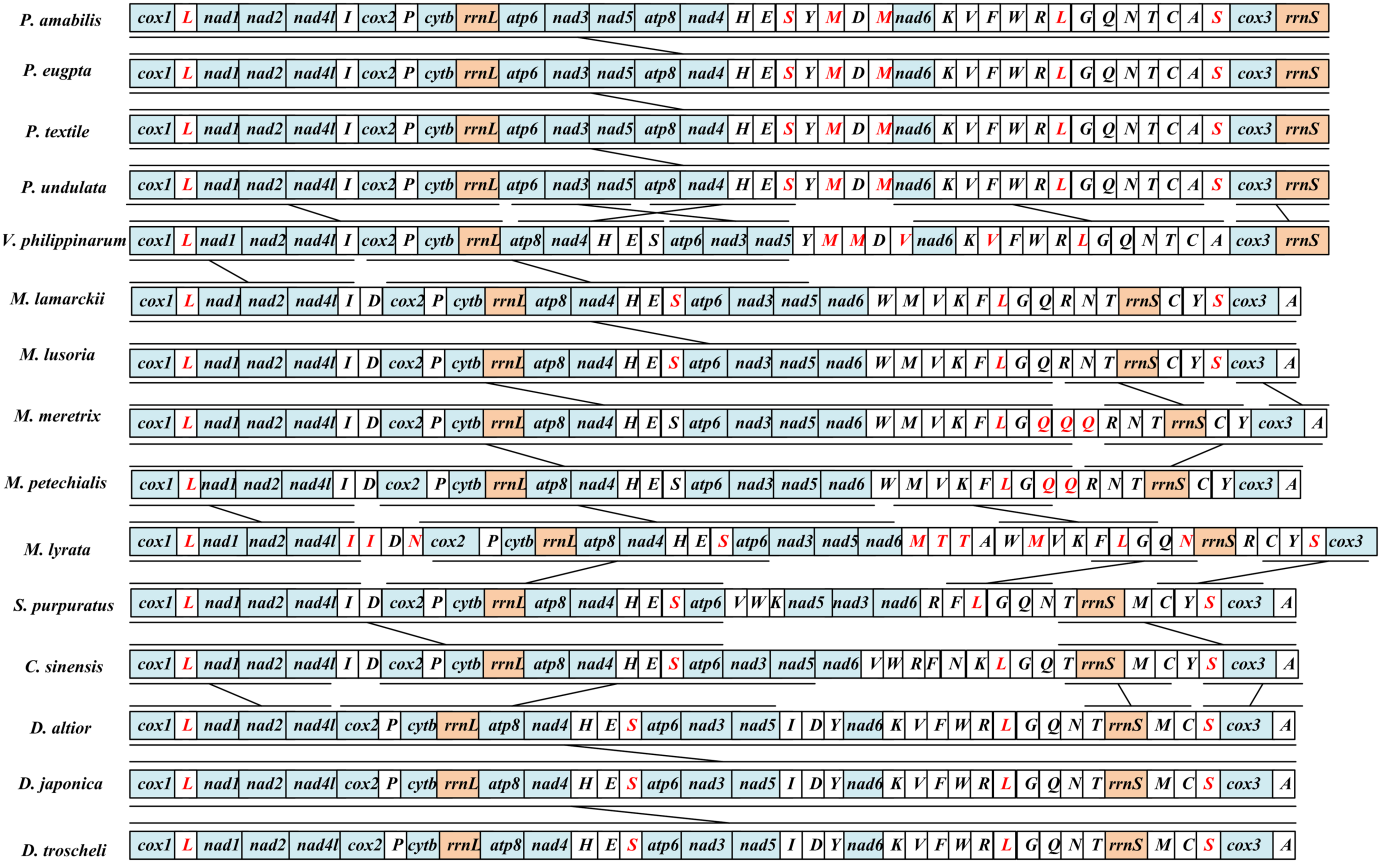
Linearized representation of gene arrangement for Veneridae mitogenomes. (All genes are transcribed from left to right. The bars indicate identical gene block. Arrows denote gene translocations, and the non-coding regions are nor presented.)

Gene arrangements appear to be dramatically variable in the major groups of bivalves, even with differences in the same family or genus [19, 28, 29]. In this study, we compare the gene arrangement of three *Dosinia* mt genomes with that of other closed related species belonging to Veneridae family (Fig 4). There were 15 Veneridae clams from six different genera (*Paphia*, *Meretrix*, *Ruditapes*, *Saxidomus*, *Cyclina* and *Dosinia*), and exhibit nine different gene orders.

In genus *Dosinia*, three *Dosinia* clams share the same gene order with each other among 37 genes, and at the same time, the four *Paphia* mitogenomes also have completely identical gene arrangements within genus. Unlike above two genera, there were four patterns of gene arrangement in the genus *Meretrix*, and this comparison was previously reported by Wu *et al*. [34] and Wang *et al*. [33]. *M*. *lamarckii* and *M*. *lusoria* have the identical gene order, but the other three *Meretrix* species have variable arrangement due to the tandem duplication of *trnQ*, *trnI*, *trnN*, and five different locations of tRNA genes (*trnT*, *trnA*, *trnQ*, *trnN*, *trnR*).

The tRNAs are more variable than either rRNAs or PCGs because of their secondary structure, which allow them to translocate more frequently [58, 59]. After excluding tRNA genes from the comparison, the gene arrangements among Veneridae mitogenomes tend to be relatively conserved. In this regard, Genera *Dosinia*, *Meretrix*, *Cyclina* and *Saxidomus* show the same gene order except for translocations of genes *nad3* and *nad5 in Saxidomus*; *Dosinia* and *Ruditapes* are almost identical except for translocations of genes *rrnS* and *cox3*. It is clear that *Dosinia* genus is more similar to *Meretrix* and *Cyclina* than *Paphia* in terms of gene arrangement. Furthermore, there is only one gene block (*cox1-nad1-nad2-nad4l-cox2-cytb-rrnL*) shared when comparing gene arrangement of PCGs and rRNAs in the 15 Veneridae species, and this gene block may be inherited from the common ancestor of Veneridae.

For the dramatic gene arrangement of mt genome during evolution in Veneridae, Xu *et al*. [27] ever explained the mt genomic rearrangement event among the *R*. *philippinarum*, *P*. *euglypta* and *M*. *petechialis* with the tandem duplication and random loss (TDRL) model, and then, the TDRL model also was used in the analyses of gene arrangements in five *Meretrix* clams [34]. The deduced evolutionary pathways from this model suggested that block interchange between adjacent genes might be common in the evolution of the mt genomes in venerids.

Comparative analysis of mitochondrial gene order has been proved to be a useful phylogenetic tool as reported in many other previous studies [4, 18, 48, 60–62]. In this study, the results are consistent with the conclusion from the phylogenetic analysis (see below), and support that gene order is a useful hallmark helping to clarify phylogenetic relationships within the family.

**Fig 5:**
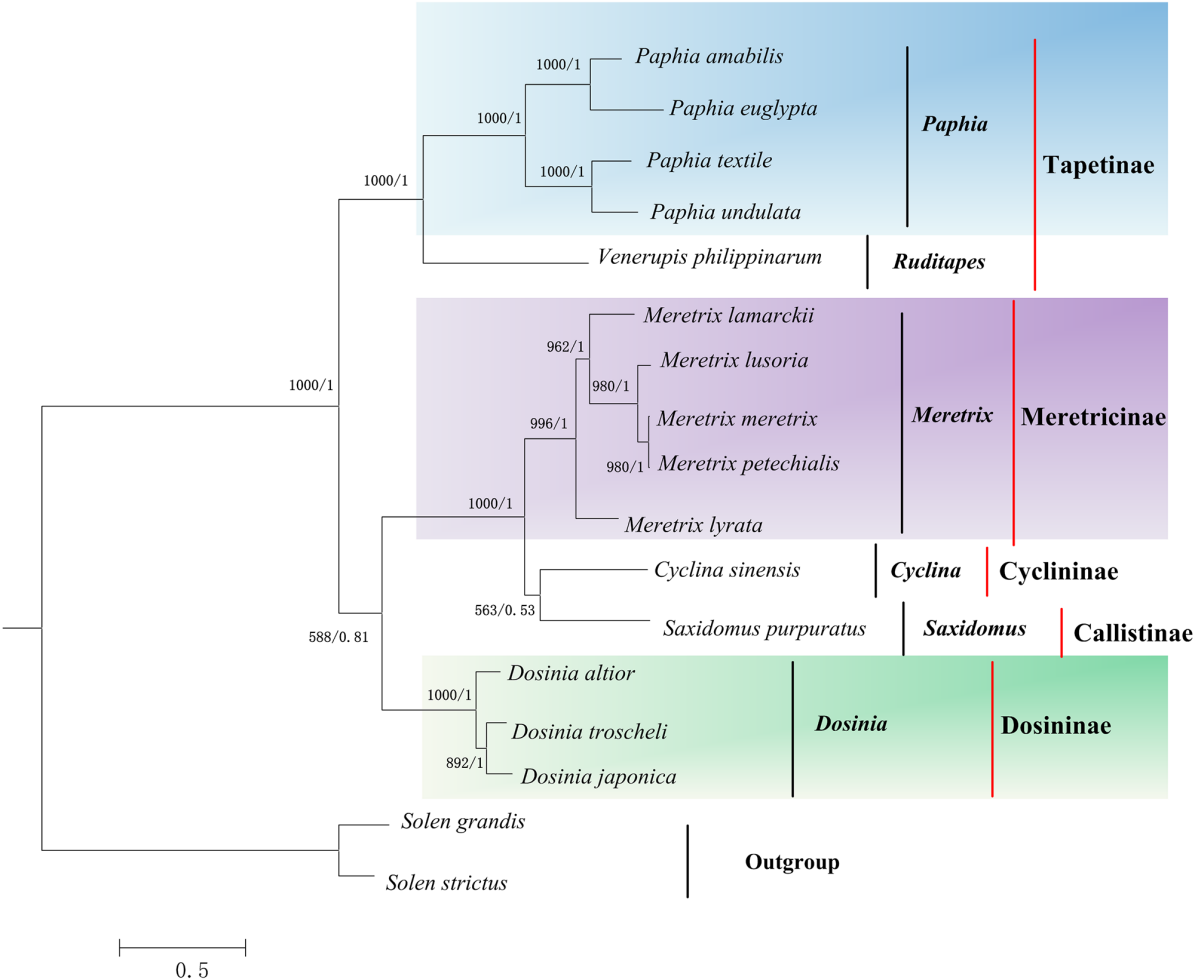
Phylogenetic trees based on the nucleotide sequences of 12 concatenated protein-coding genes. The first number at each node is Bayesian posterior probability and the second number is ML bootstrap values. The colorful parts are the genus which including more than one species except outgroup.

ML and BI trees based on the nucleotide sequences of 12 concatenated protein-coding genes (except *atp8* gene) were performed to construct phylogenetic relationships within family Veneridae (Fig 5). The topological structures showed that the results of ML and BI trees were in almost complete agreement.

The phylogenetic analysis indicated that three species belonging to genus *Dosinia*, including *D*. *japonica*, *D*. *altior* and *D*. *troscheli* clustered together, supporting that *Dosinia* is monophyletic, which was in accordance with previous viewpoints [6, 7]. At the same time, *D*. *japonica* and *D*. *troscheli* formed a single group at the *Dosinia* level, with *D*. *altior* being the sister taxon. This result is consistent with the morphological classification [35], in which *D*. *altior* was classified into the subgenus *Bonartemis*, *D*. *japonica* and *D*. *troscheli* were classified into another subgenus *Phacosoma*.

In this study, according to the phylogenetic tree, Dosininae and Meretricinae have a closer relationship, with Tapetinae being the sister taxon, which indicates Dosininae and Meretricinae may have a common ancestor based on the nucleotide sequences. Our results were in agreements with the previous studies discussing the phylogenetic relationships which including Veneridae family within Heterodont or Veneroida [3, 13, 14, 16, 18]. Besides, there is a disagreement about the sister relationship between Dosininae and Meretricinae, which ever reported by Chen *et al*.[6] and Lv *et al*.[10] based on short fragments of nuclear gene or mt DNA. This similar phenomenon had also happened to Cardioidea and Tellinoidea which was reported by Yuan *et al*. [18], and different evolutionary position of *M*. *lyrata* and *M*. *lamarckii* by Wu *et al*. [34] and Fernandez-Perez *et al*. [4]. The credibility of the results in this study include: i) the number of mt genome was increased to enhance the accuracy of evolutionary position, avoiding only one mitogenome of *Dosinia* considerable divergence from the other venerids; ii) mt genome contains more genetic information than short gene fragments, and short gene fragments existed the substitutional saturation of nucleotide and horizontal gene transfer; and iii) this conclusion was in accordance with the analyses of gene arrangement among 15 mt genomes in Veneridae family in this study.

The phylogenetic analysis here provided a real possible phylogenetic relationship among *Dosinia* species and their evolutionary position within family Veneridae, and in this sense, results of phylogenetic analyses support the idea that genome reorganization among congeneric species is not random, but is correlated with their phylogenetic relationships. In future, more detailed analyses with a larger taxon sampling and more rapidly evolving molecular markers including mt genome are still necessary in order to clarify the phylogenetic relationships within family Veneridae.

## Conclusion

In this study, we sequenced the complete mt genomes of *D*. *altior* and *D*. *troscheli*, and compared the genus-specific genomic features of mitogenomes in *Dosinia* genus. Our study has provided insights into the evolutional relationships among Veneridae clams. Based on phylogenetic analyses of multiple protein-coding genes, we suggest that *Dosinia* genus would be evolutionarily closer to Meretricinae clams than Tapetinae, and support that the *Dosinia* is monophyletic. Nevertheless, additional analyses with mitochondrial genomic information of more diverse clam species will be required to further understand the phylogenetic relationships in family Veneridae including genus *Dosinia*.

## Supporting information

S1 Fig Putative secondary structure of the 22 tRNAs predicted based on the *Dosinia altior* mitogenome sequence. (TIF)

S2 Fig Putative secondary structure of the 22 tRNAs predicted based on the *Dosinia troscheli* mitogenome sequence. (TIF)
