# Supplementary figures and images for: Comparative analyses of the complete mitochondrial genomes of Dosinia clams and their phylogenetic position within Veneridae
Source: PLoS One. 2018 May 2;13(5):e0196466. doi: 10.1371/journal.pone.0196466 (PMC5931646; doi:10.1371/journal.pone.0196466)

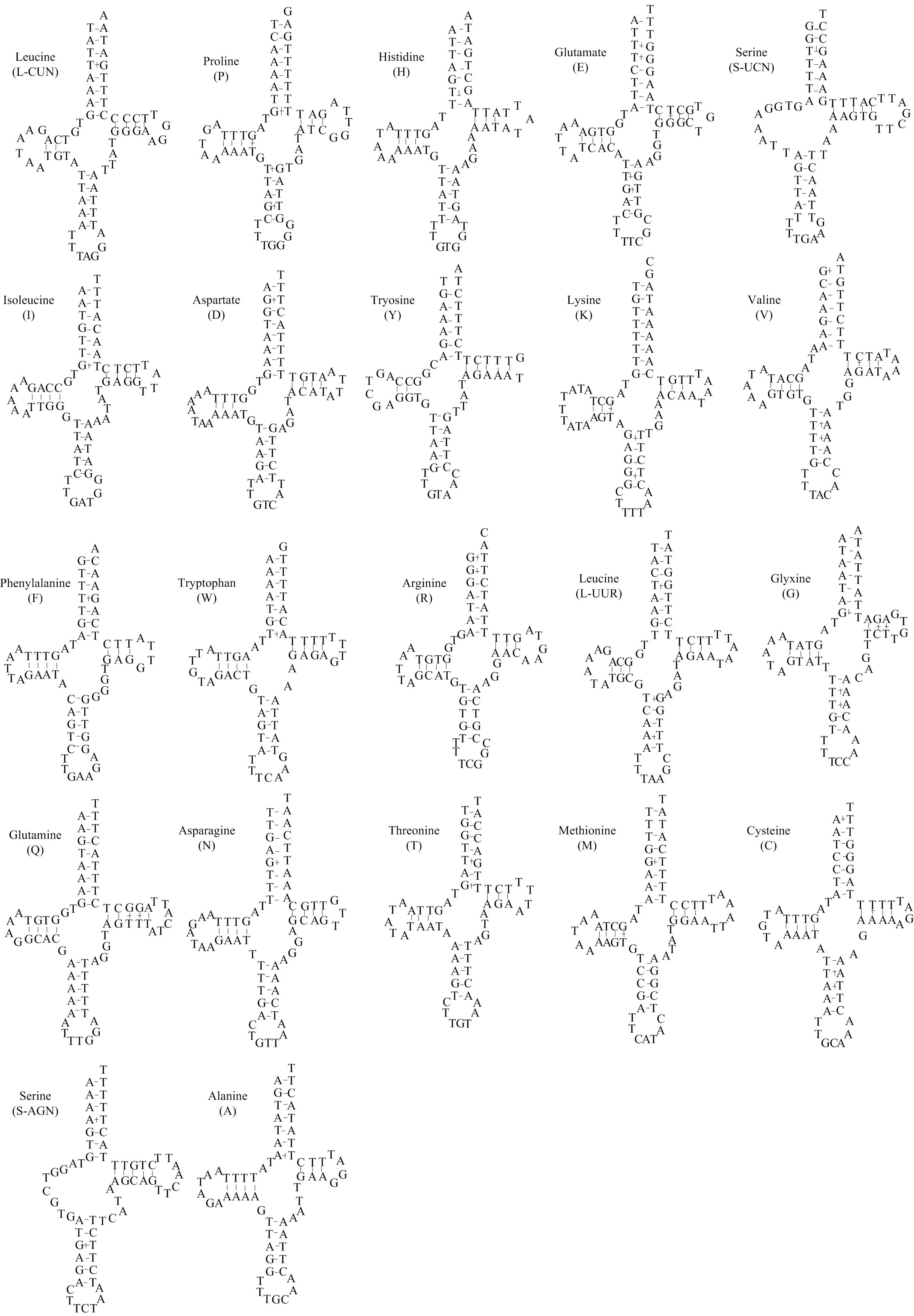

Supplement: S1 Fig — (TIF) [file pone.0196466.s001.tif]

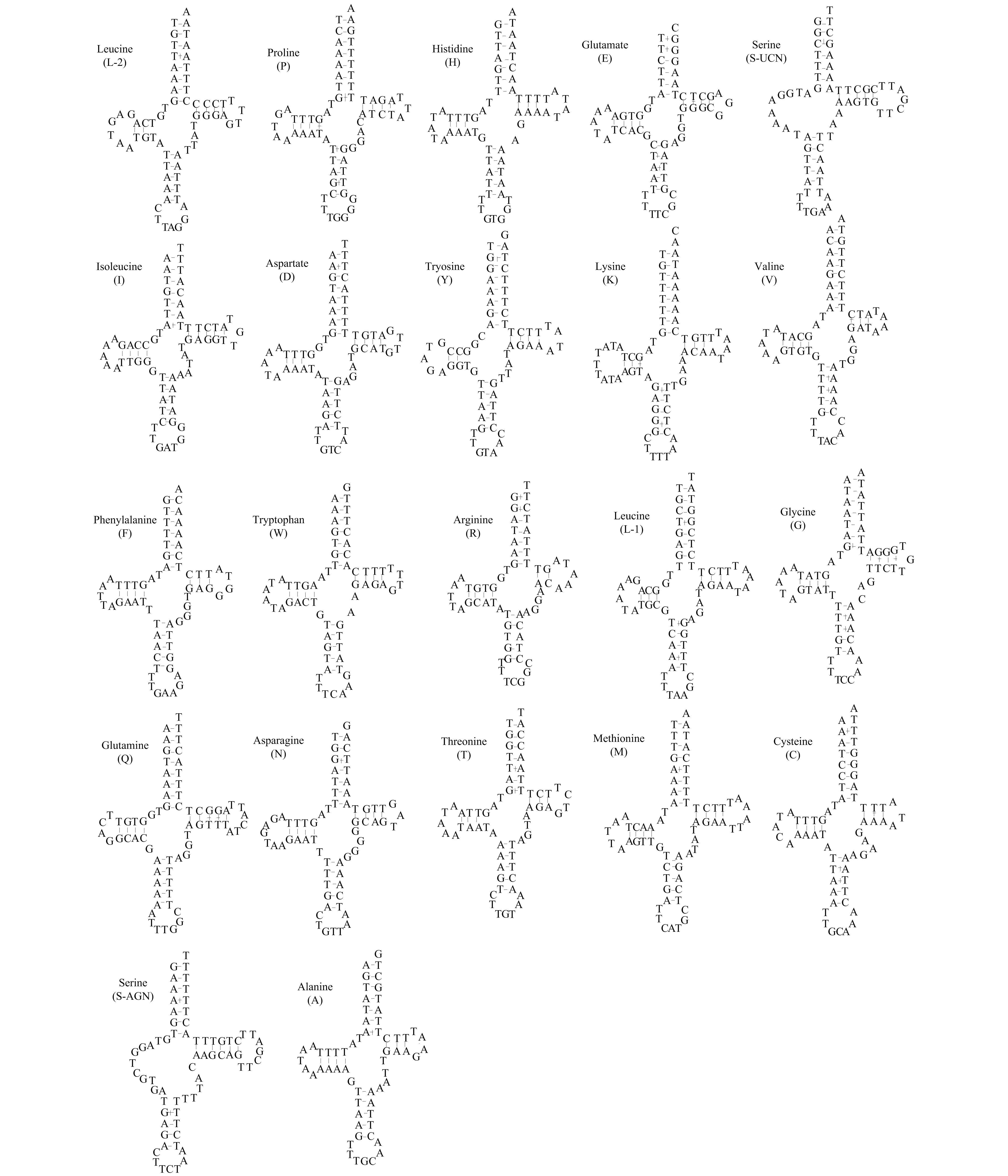

Supplement: S2 Fig — (TIF) [file pone.0196466.s002.tif]
